# [Corrigendum] MicroRNA-182 promotes epithelial-mesenchymal transition by targeting FOXN3 in gallbladder cancer

**DOI:** 10.3892/ol.2026.15701

**Published:** 2026-06-12

**Authors:** Jianhong Zhang, Zeming Hu, Chao Wen, Qicheng Liao, Baoqing He, Jing Peng, Xin Tang, Zhixi Chen, Yuankang Xie

Oncol Lett 21: 200, 2021; DOI: 10.3892/ol.2021.12461

Subsequently to the publication of the above paper, an interested reader drew to the authors’ attention that, concerning the cell invasion and migration assay experiments shown in Fig. 6A on p. 8, the ‘Invasion/miR-NC’ data panels for the GBC-SD and SGC-996 cell lines showed a small overlapping section, such that these data appeared to have been derived from the same original source where the results from the two different cell lines were intended to have been portrayed. In addition, for the immunoblot assay experiments shown in Fig. 6H and I, the same data had apparently been included in this figure to show the results of the IP: IgG - IB: FOXN3 experiments for the two cell lines. Furthermore, upon performing an independent analysis of the data in this paper in the Editorial Office, it came to light that certain of the western blot data shown in Fig. 5C and I, and immunoblot assay data in Fig. 6H and I, had subsequently reappeared in three other published papers that featured several of the authors listed above in common (doi: 10.7150/jca.66850; doi: 10.1080/ 21655979.2022.2079302, and doi: 10.3389/fphar.2021.680585), with the co-author Zhixi Chen being an author on all the papers;

The authors were able to re-examine their original data, and realized that certain of the data included in Fig. 6A, H and I were presented erroneously; to rectify the issue of the re-used western blot data among the four papers mentioned above, the authors have repeated the experiments shown in Fig. 5C and I. The revised versions of Fig. 5 (showing the replacement data for Fig. 5C and I, where the experimental results were found to be broadly similar to the data shown in the original figure) and Fig. 6 (now featuring replacement data for the invasion assay experiments in Fig. 6A for the GBC-SD and SGC-996 cell lines, and replacement immunoblot data for Fig. 6H and I) are shown on the next two pages. The authors regret the errors that were made while compiling the original figures, and are grateful to the editor of *Oncology Letters* for allowing them the opportunity to publish this Corrigendum. Note that the errors in this pair of figures did not have a significant impact on the conclusions reported in this study. All the authors agree with the publication of this corrigendum; furthermore, they apologize to the readership for any inconvenience caused.

## Figures and Tables

**Figure 5.**
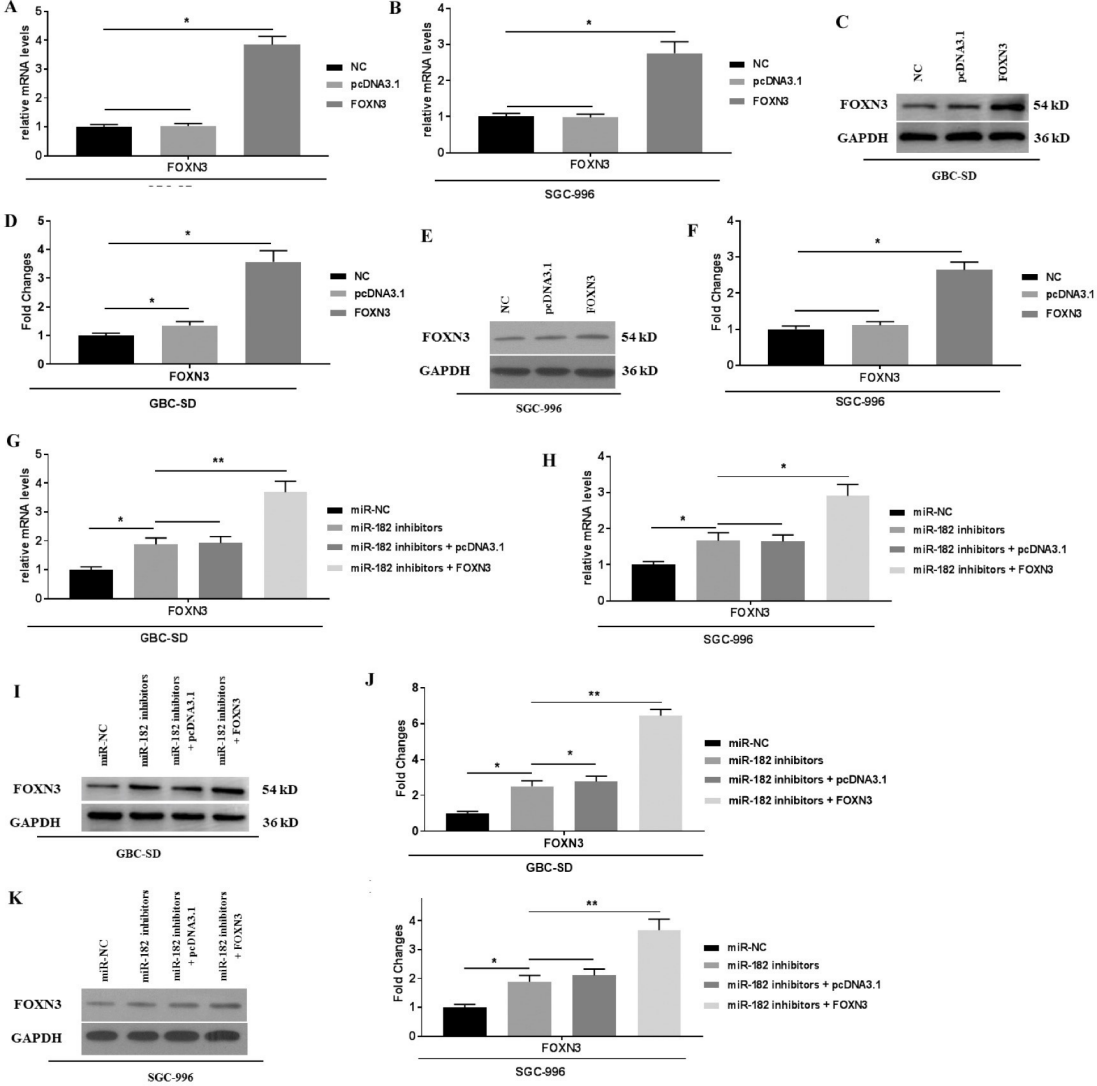
Co-transfection with FOXN3 and miR-182 inhibitors into GBC cells. Reverse transcription-quantitative PCR analysis was performed to detect mRNA FOXN3 expression in (A) GBC-SD and (B) SGC-996 cells transfected with pcDNA3.1-FOXN3. Western blot analysis was performed to detect protein FOXN3 expression in (C and D) GBC-SD and (E and F) SGC-996 cells transfected with pcDNA3.1-FOXN3. Similarly, the mRNA FOXN3 expression in (G) GBC-SD and (H) SGC-996 cells co-transfected with pcDNA3.1-FOXN3 and miR-182 inhibitors. The protein FOXN3 expression was also determined in (I and J) GBC-SD and (K and L) SGC-996 cells co-transfected with pcDNA3.1-FOXN3 and miR-182 inhibitors. All experiments were performed in triplicate and data are presented as the mean ± standard deviation. *P<0.05 and **P<0.01. FOXN3, Forkhead box N3; miR, microRNA; GBC, gallbladder cancer; NC, negative control.

**Figure 6.**
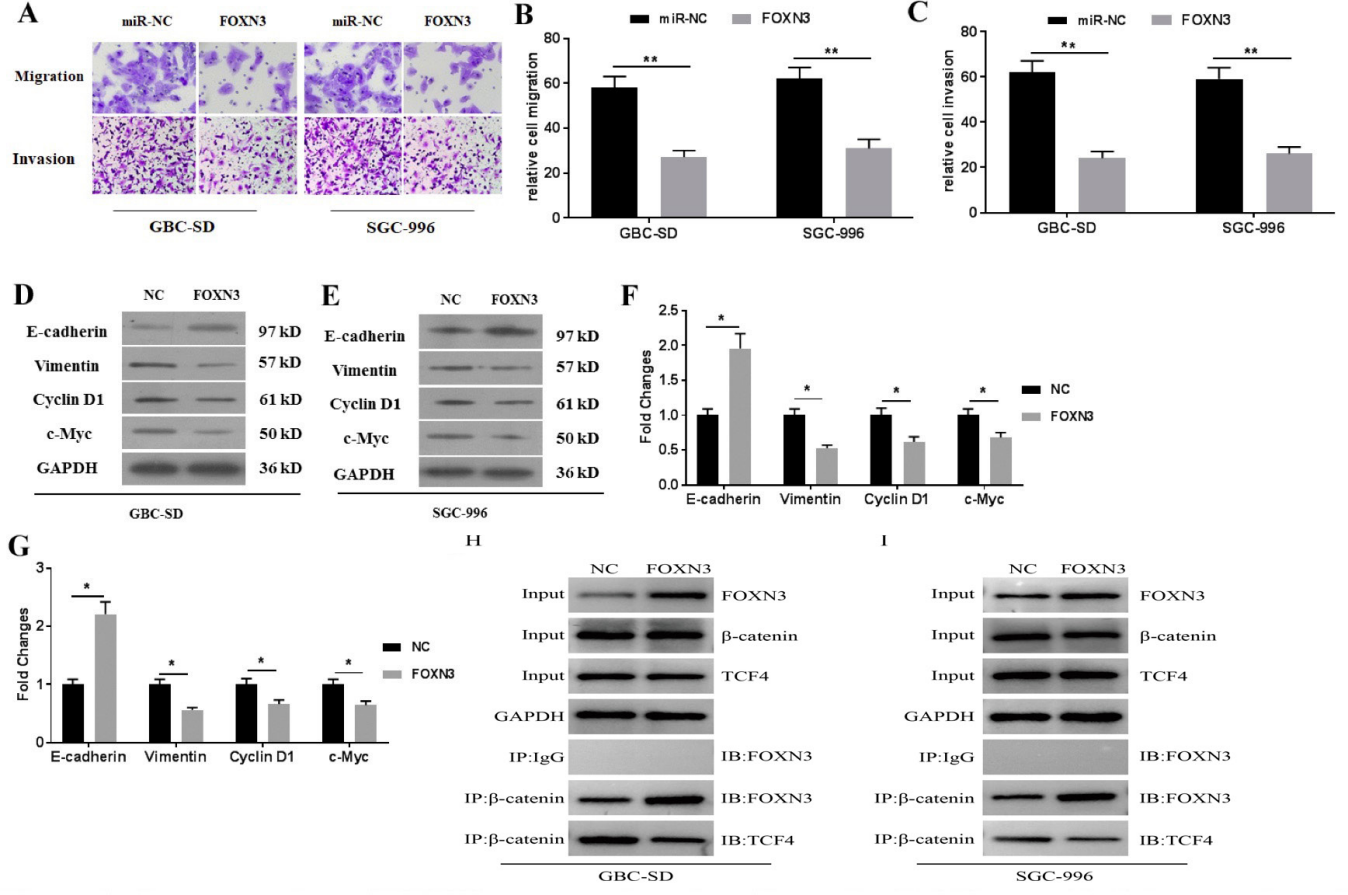
Overexpression of FOXN3 compromises the effects of miR-182 on epithelial-mesenchymal transition in GBC cells co-transfected with miR-182 inhibitors and pcDNA3.1-FOXN3. (A) The cell migratory and invasive abilities were detected via the Transwell assays following transfection with miR-NC and FOXN3, respectively. (B) The summarized staining levels for migration. (C) The summarized staining levels for invasion. Western blot analysis was performed to detect the protein expression levels of E-cadherin, Vimentin, Cyclin D1 and c-Myc in (D) GBC-SD and (E) SGC-996 cells. (F) The summarized data of (D). (G) The summarized data of (E). (H and I) The Co-IP assay was performed to assess the association between FOXN3 and β-catenin. Cell protein extracts (10%) were used as the input sample, which was subjected to Western blot analysis. The remaining protein extracts were subjected to IP using control goat IgG or β-catenin antibodies, followed by IB with anti-FOXN3 or anti-TCF4. All experiments were performed in triplicate and data are presented as the mean ± standard deviation. *P<0.05 and **P<0.01. FOXN3, Forkhead box N3; miR, microRNA; GBC, gallbladder cancer; NC, negative control; IP, immunoprecipitation; IB, immunoblotting.

